# Flexible and Stretchable Bio-Integrated Electronics Based on Carbon Nanotube and Graphene

**DOI:** 10.3390/ma11071163

**Published:** 2018-07-08

**Authors:** Taemin Kim, Myeongki Cho, Ki Jun Yu

**Affiliations:** School of Electrical Engineering, Yonsei University, Seoul 03722, Korea; taeminkim@yonsei.ac.kr (T.K.); mango_cho@yonsei.ac.kr (M.C.)

**Keywords:** flexible electronics, carbon-based nano-materials, bio-integrated electronics

## Abstract

Scientific and engineering progress associated with increased interest in healthcare monitoring, therapy, and human-machine interfaces has rapidly accelerated the development of bio-integrated multifunctional devices. Recently, compensation for the cons of existing materials on electronics for health care systems has been provided by carbon-based nanomaterials. Due to their excellent mechanical and electrical properties, these materials provide benefits such as improved flexibility and stretchability for conformal integration with the soft, curvilinear surfaces of human tissues or organs, while maintaining their own unique functions. This review summarizes the most recent advanced biomedical devices and technologies based on two most popular carbon based materials, carbon nanotubes (CNTs) and graphene. In the beginning, we discuss the biocompatibility of CNTs and graphene by examining their cytotoxicity and/or detrimental effects on the human body for application to bioelectronics. Then, we scrutinize the various types of flexible and/or stretchable substrates that are integrated with CNTs and graphene for the construction of high-quality active electrode arrays and sensors. The convergence of these carbon-based materials and bioelectronics ensures scalability and cooperativity in various fields. Finally, future works with challenges are presented in bio-integrated electronic applications with these carbon-based materials.

## 1. Introduction

Over the past few decades, commercial nanoscale electronic devices, based on semiconductor wafers composed of single-crystal inorganic materials, have achieved high performance with a significant development of fabrication processes. Despite these technological advances, devices with bulky, rigid, and planar materials have limitations for use as flexible or stretchable electronics [1,2,3] because of their poor mechanical properties, such as high Young’s modulus and mechanical rigidity [4], which can easily induce device fracture when it comes under conditions of large mechanical deformation. To resolve this problem, the establishment of soft–hard integrated materials [5,6] or the integration of ultra-thin semiconducting materials with soft substrates are strongly suggested for flexible and stretchable electronics [7,8,9,10]. Recently, studies on flexible and stretchable electronic devices have demonstrated various electronic applications, such as passive/active circuit elements (e.g., resistor, diode, and transistor), sensors (e.g., strain, temperature, and electrochemical sensor), wireless radio-frequency (RF) communication components (e.g., capacitor, inductor, oscillator, and antenna), and light-emitting elements (e.g., organic light-emitting diode) on substrates with various materials [11,12,13,14]. Furthermore, the flexible or stretchable mechanical properties of these devices offer potentially unique opportunities in bio-electronic applications for health monitoring and treating diseases [15,16,17,18,19,20,21], mainly because of their ultrathin profiles that eliminate the mechanical mismatch between the tissue or organs and electronics. The tissues of the human body have soft, curvilinear surfaces and even have dynamic movements, such as the swelling and contraction of heart [22,23]. Flexible or stretchable electronics on polymer-based substrates [24,25,26,27,28] (e.g., parylene, SU8, and polyimide), which reduce the mismatch between the device and the organs, provide the possibility for conformal, intimate integration with tissues for advanced healthcare from the measurement of electrophysiological signals [29], delivery of drugs or stimulations [30], to well-established human-machine interfaces [21,31]. Recently, carbon-based nanomaterials, such as graphene and carbon nanotubes (CNTs), have been applied in the field of bioelectronics because of their extraordinary mechanical properties, and unique electrical and optical properties based on their inherent geometric structures [32,33,34,35]. Both graphene and CNTs show similar properties, except for their physical structures. Particularly, the extraordinary mechanical properties of these materials, such as an approximately 1 TPa Young’s modulus and 25% fracture strain [36,37], along with their subnanometer thicknesses are highly advantageous when manufacturing flexible and stretchable bioelectronics for conformal contact with tissues. Furthermore, graphene and CNTs are suitable materials for use in bioelectronics, because they have high stability and low biofouling in biological environments as well as high reproducibility with direct growth [38,39,40,41,42]. In this review, we introduce some of the key flexible electronics with carbon-based materials that can be utilized not only for intrinsically conformal contact with tissues, but also for biocompatibility in physiological environments for applications in bio-integrated electronics.

## 2. Biocompatibility

Using solely biocompatible materials is one of the most significant undertakings in biomedical systems, especially for epidermal electronics [5,11] and implantable electronics [15,17,20,43,44]. Before soft materials are integrated into existing or new novel bioelectronics, toxicity, such as cytotoxicity, and the immune system response to these materials must be precisely examined. Previous studies about the toxicity of carbon nanomaterials in the human body have been reported [45,46,47,48]. However, the effects on the long-term usage of these materials in biological environments has not been clearly carried out yet. Therefore, we are still required to understand the potential toxicity of carbon nanomaterials for long-term implants.

Over the last few decades, carbon-based materials such as pyrolytic carbon (PC) and diamond-like carbon (DLC) have been used in the biomedical field. PC has been used in biomedical implants and coating materials, especially in the production of heart valve prostheses [49]. Most PCs are anisotropic in nature. By advancing the manufacturing using chemical vapor deposition (CVD) processes, isotropic PC coatings on heart valves have been achieved [50]. PC heart valves show excellent biocompatibility, including good blood compatibility, good adhesion to endothelial cells, and minimal adhesion to inactive platelets [51,52,53]. More recently, DLC has been developed more than PC as a biomedical carbon-based material. Via postprocessing DLC coating, properties such as hardness, surface lubrication, and insolubility can be obtained through carbon-based nanomaterials, along with biocompatibility [54,55,56,57]. Conventional carbon-based biomedical materials have been successfully commercialized because of their superior biocompatibility. Herein, we discuss the specific biocompatibility of graphene and carbon nanotubes, which can be extensively used as devices in biomedical engineering or human healthcare systems.

CNTs have always been controversial in terms of biocompatibility. These conflicting arguments are influenced by the effects of the impurities in CNTs, the length of CNTs, surface chemistry, dispersion interaction between various factors, and experimental variables, including different doses of CNTs, cell population, and evaluation systems [58]. CNTs are a type of nanoparticle that is detrimental to the body when inserted into the body, because of their high surface area and surface toxicity. Recent studies on CNT nanoparticles associated with biocompatible materials address the toxicity in lungs, skin irritation, and cytotoxicity. Asbestosis, lung cancer, and pleural mesothelioma can be triggered by CNT inhalation [59,60,61]. As the CNT resembles asbestos, which has the fibrous structural characteristics, CNTs are regionally deposited in the lung and cause the diseases [62,63]. Brown et al. reported that the release of the pro-inflammatory cytokine tumor necrosis factor (TNF-α) and the reactive oxygen species is increased in the monocytic cells exposed to nanotubes, and frustrated phagocytosis is observed, which could inhibit the elimination of nanotubes from the lungs by macrophage [64]. However, the pathway of CNT nanoparticles into the lungs without inhalation has not been clearly eluded. There are previous studies on skin irritation caused by CNT [65,66], however, Ema et al. evaluated dermal and eye irritation and skin sensitization experiments in rabbits and guinea pigs and demonstrated Nikkiso-single-walled carbon nanotubes (SWCNTs), super-growth SWCNTs, and the Mitsui product of multi-walled carbon nanotubes (MWCNTs) were not irritants to the skin or eyes [67]. The cytotoxicity of CNT has been more broadly researched. In the past, the cytotoxicity of CNTs in mammalian cells has been examined in various types of in vitro cells [65,66,68,69]. In contrast, some reports have determined noncytotoxic CNT in mammalian cells [70,71,72,73]. Depending on the degrees of aggregation of the CNTs, the physical shapes and surface areas of CNTs can be formed differently, which leads to different effects of cytotoxicity. Belyanskaya et al. investigated the effects of differently agglomerated SWCNTs on neural cells from the central and peripheral nervous system of the chicken embryo. The results showed that the more agglomerated SWCNTs are the more toxic, by decreasing the overall DNA content [74]. Wick et al. demonstrated that well-dispersed CNTs are less cytotoxic, and rope-like agglomerated CNTs are more cytotoxic than asbestos on human MSTO-211H cells [75]. On the other hand, however, Mutlu et al. reported that highly dispersed SWCNTs show nontoxicity in vitro or in vivo [76]. Also, Kolosnjaj-Tabi et al. demonstrated no granuloma formation when the length of the CNTs, which are intraperitoneal injections, of mice was under 10μm [77]. On the side of the CNTs over the substrate, not the particles or fiber, there are many reports about CNT-based biomedical materials with their acceptable biocompatibility. Elias et al. examined carbon nanofiber compacts for biomaterials because of their unique adhesion with bone-forming osteoblast cells, and determined the biocompatibility for their usage as orthopedic or dental implantable materials [78]. Similarly, Lobo et al. demonstrated the biocompatibility of vertically aligned MWCNT films by culturing L929 mouse fibroblast cells [79]. The cell adhesion and morphology of the L929 mouse fibroblast cells were observed after seven days using scanning electron microscopy. The cells from the first layer spread out and survived for seven days by clearly showing a good cytocompatibility of aligned MWCNT to the fibroblast cells. In neuronal growth studies, chemically functionalized multi-walled carbon nanotubes (MWCNTs), especially neurons grown on MWNTs with positive charge, showed the excellent neurite outgrowth and good biocompatibility [80].

The chemical composition and crystalline structure of both graphene and CNTs are similar. However, some properties of these two materials are different, because the two-dimensional (2D) structure of graphene is fundamentally different from the cylindrical structure of CNT. These materials have different mechanisms of interaction with cell systems, resulting in definite differences in the toxicity of living cells between graphene and CNT. Therefore, the biocompatibility of graphene should be carefully examined separately from CNTs. Recent studies on dispersed graphene flakes using a liquid-phase exfoliation (LPE) [81] of graphite to obtain biocompatible graphene flakes, have been reported [82,83,84]. Castagnola et al. used a complete serum to disperse graphene for the exploration of biological interactions and resolved the protein composition on the dispersed graphene nanoflakes [85]. The results showed that most of the proteins, except for apolipoprotein B100, in a complete serum have good affinity, which proves the biological identity of the graphene nanoflakes. However, in a real complex biological environment, the evaluation of an interaction between the dispersed graphene flakes and proteins is complicated and should be considered more carefully. On the other hand, some research repeatedly reported that the graphene sheet interacts with protein molecules and causes a disruption to the structure and function of proteins. The graphene sheet disrupted the α-helical structures of the peptides by interacting with the villin headpiece (HP35) protein [86,87]. The distortion in the villin headpiece (HP35) proteins proceeds with almost losing the α-helical elements, which are adsorbed to the graphene surface, because of the π–π stacking interaction between the graphene lattice and the aromatic residues. Zhao et al. demonstrated that graphene also disrupted the double stranded DNA segments [88]. In an aqueous solution, the self-assembly phenomenon between the DNA segments with graphene were observed. Some of the DNA are vertically standing while others are horizontally laying on the graphene surface. The latter case of the DNA segments, which were damaged because of several mutations, leads to DNA toxicity. There are studies of graphene with regards to biocompatibility. Sahni et al. presented a great biocompatibility of graphene by culturing rat neuronal cells grown on graphene-coated surfaces [89]. The results verified the biocompatibility of graphene surfaces with direct neuronal interfaces. As another example, Kalbacova et al. demonstrated that human osteoblasts and mesenchymal stromal cells can be cultured on monolayer graphene films [90]. The results showed that, compared to that of cells that were cultured on a silicon oxide substrate, the adherence and proliferation of the cells cultured on the graphene were better, and no cytotoxicity for human osteoblasts and mesenchymal stromal cells was demonstrated. Furthermore, Li et al. also successfully cultured neural stem cells on three-dimensional graphene foams (3D-GFs) [91]. The cytotoxicity of the 3D-GFs was estimated by calcein acetoxymethyl ester (calcein-AM) and EthD-I staining assays, with a 2D graphene film as the control. There was no difference in the cell viability between the 3D-GFs and 2D graphene film five days after culturing without abnormal cell apoptosis on 3D-GFs. In addition, Bendali et al. demonstrated the excellent cytocompatibility of single-layer graphene by directly growing primary adult retinal ganglion cells on a graphene surface without any peptide coating [92]. This work differs from the existing studies in its use of purified adult and differentiated retinal neurons instead of stem cells.

## 3. CNT/Graphene Based Microelectrodes for Neural Interface

### 3.1. CNT-Based Microelectrodes

CNTs are considered promising materials for flexible neural interfaces because of their high conductivity and mechanical strength. The electrophysiological signals such as electroencephalogram (EEG) or electrocorticogram (ECoG) are directly affected by the impedance of the electrodes, which lead to the extremely low current injected into the electrodes [93]. High impedance gives rise to more interference of the thermal noise that, as a result, decreases the sensitivity towards the signal. Hence, lowering the impedance by increasing the effective contact area of the electrodes has been prime importance in order to measure the more reliable action potentials with minimal noise. However, the low impedance and high spatial resolution of neural electrodes are in a tradeoff relationship. As the ultimate goal of the neural interfaces is to selectively measure the action potential of every single neuron, increasing the size of the electrodes is contrary to this goal. Therefore, it is attracting great attention as a method to increase the sensitivity while maintaining a high selectivity, by replacing conventional planar electrodes with a variety of nanomaterials or by making nanostructured surfaces using such nanomaterials [94,95,96] to achieve a higher contact areal-density in a unit area.

**Figure 1:**
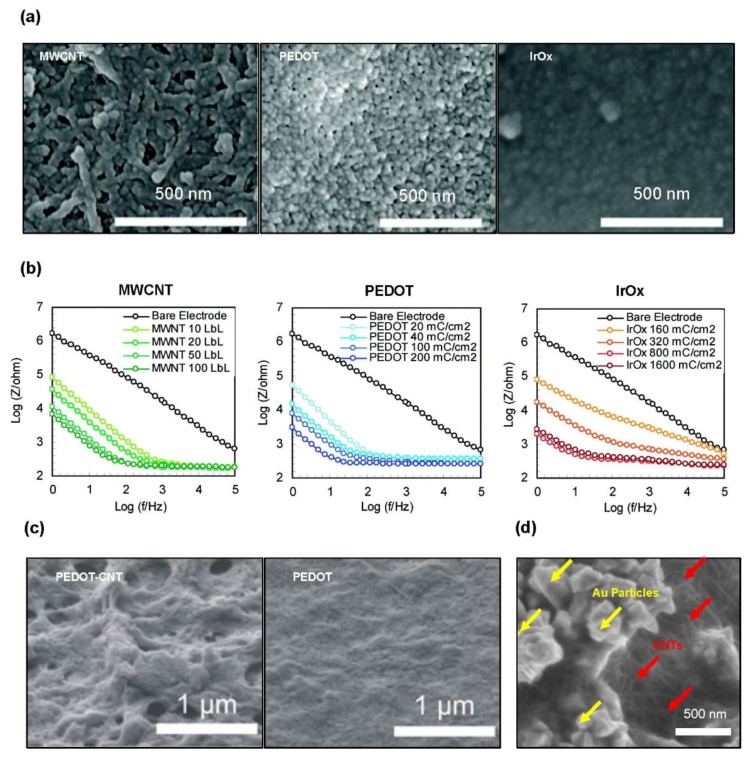
Surface texturing by coating with nanomaterials to lower the electrochemical impedance: (**a**) SEM images of PtIr electrodes coated with multi-walled carbon nanotubes (MWCNTs) (left), poly(3,4-ethylenedioxythiophene) (PEDOT) (middle), and IrOx (right); (**b**) electrochemical impedance spectroscopy (EIS) of three surface-modified electrodes with the increasing film thicknesses over a frequency range of 1–105 Hz. Reproduced with permission from reference [100], Copyright 2009, American Chemical Society; (**c**) SEM images to show the surface morphology of the PEDOT-carbon nanotubes (CNTs) composite (left) and pure PEDOT (right) coatings. Reproduced with permission from reference Gerwig et al. [102], Copyright 2012, Frontiers; (**d**) SEM image of an electrodeposited Au-CNT composite network. Reproduced with permission from reference Xiang et al. [103], copyright 2014, IOP Publishing Ltd.

CNT-based microelectrodes have much higher surface areas than those of flat surface electrodes in the same planar surface as a result of the unique one-dimensional (1D) geometry of CNTs (Figure 1), which gives three-dimensional (3D) space to the surface. This high surface area combined with the inherently outstanding conductivity and stability [97,98,99] makes CNTs promising as active nanomaterials for highly sensitive neural interfaces. Jan et al. coated three PtIr electrodes with MWCNT, synthesized by layer-by-layer (LBL) deposition, poly(3,4-ethylenedioxythiophene) (PEDOT), and IrOx, respectively, and compared their electrochemical properties to electrochemical impedance spectroscopy (EIS) and cyclic voltammetry (CV) [100]. As shown in the SEM image and the EIS graph, in Figure 1a,b, the roughness of the surface decreases in the order of MWCNT, PEDOT, and IrOx, and the impedance increases in the same order. The thickness of the MWCNT LBL coating increases by 7 nm with the addition of bilayers, resulting in 700 nm at 100 LBL. At a 700 nm coating thicknesses among the various electrodes, the MWCNT LBL electrodes show 30% and 60% lower impedances than those of the PEDOT and IrOx electrodes, respectively, at 1 kHz.

Similar to CNTs, PEDOT, well known as a representative conducting polymer, has also been regarded as a biocompatible sensing material that provides improved selectivity and enables further surface modification [101]. The electrochemical properties of PEDOT-coated electrodes can be further enhanced when fabricated with CNTs, while maintaining high mechanical flexibility. Gerwig et al. demonstrated that the surface roughness of the PEDOT-CNT composites was higher than that of the pure PEDOT, because of the porous morphology (Figure 1c) [102]. This increased surface roughness, as well as the CNTs acting as a conducting network, provides improved impedance spectra. Figure 1d shows an additional example of a CNT coating, where the CNTs are mixed with conventional metal particles rather than soft materials, so as to increase the effective surface area [103]. Compared with the bare Au contacts, the CNT-Au composite contacts offered lower electrochemical impedance over a wide band of the frequency range from 10 Hz to 100 kHz; in particular, the impedance was 18 times lower at 1 kHz. The ultrathin neural probe with these CNT-Au composite electrodes successfully recorded the neural activity from the rat’s hippocampus CA1 area, with a high signal-to-noise ratio (SNR). 

Electrophysiological signals recorded using novel microelectrodes provide an excellent temporal resolution, but the optical imaging of neural circuits must be followed to achieve both high temporal and spatial resolution [104,105,106]. Furthermore, unlike electrical stimulation, the advent of optogenetics, which can simultaneously perform electrophysiological readout and stimulation, has led to additional demand for transparent devices [107,108]. However, conventional opaque electrodes and interconnects preclude the optical imaging and stimulation of tissues just below the site, where those components are located, and produce light-induced artifacts that contaminate the electrical recording [109]. These challenges limit the use of metal electrodes, such as Pt or stainless steel, which exhibit higher electrical conductivity than that of CNTs and graphene. Even though indium-tin oxide (ITO) is widely used as a transparent conductor because of its high transmittance in the visible light region, it is difficult to use in bio-integrated electronics, because of its poor mechanical flexibility, which induces fractures under large deformations in the neural surface. 

**Figure 2:**
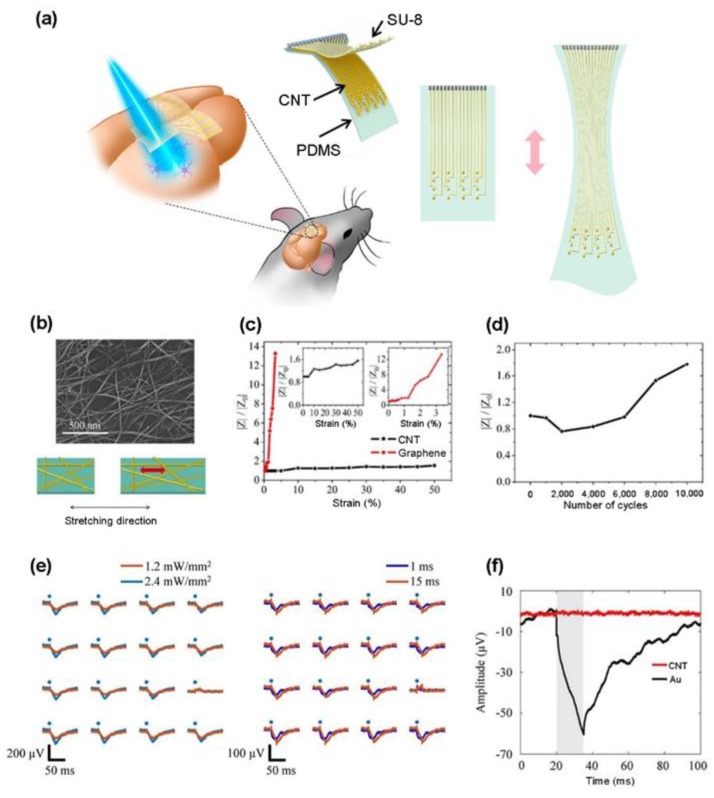
CNT-based transparent electrode arrays for simultaneous electrical recording and optical stimulation: (**a**) schematic illustrations of the stretchable transparent CNT electrode array and its application for optogenetics; (**b**) an SEM image of the CNT network thin film (top) and a schematic illustration of the CNT network thin film under stretching (bottom); (**c**) the strain-dependent impedance change at 1 kHz of the CNT and graphene electrodes under the applied tensile strain; (**d**) impedance change at 1 kHz of a CNT electrode during 10,000 stretching cycles with 20% strain; (**e**) the mapping of evoked potentials after optical stimulations under different stimulus intensity (left) and duration (right). Blue dots indicate the stimulus time points; and (**f**) the light-induced artifacts in CNT and Au electrodes under a photostimulus (2.4 mW/mm^2^, 15 ms). The gray box indicates the duration of stimulation pulses. Reproduced with permission from Zhang et al. [110], copyright 2018, American Chemical Society.

Here, we introduce recent advances in transparent and flexible microelectrode arrays, based on CNTs and graphene for simultaneous electrical and optical interaction with brain tissues. Figure 2a shows an example of the transparent and stretchable microelectrode array from CNT thin films fabricated by Zhang et al. for optogenetic stimulations with simultaneous ECoG recording [110]. The stretchable transparent electrode array with these films integrates an elastic PDMS substrate, CNT electrodes and interconnects, and SU-8 insulation layer, retaining a high optical transmittance across a broad wavelength range of 400 nm to 2.5 μm. The CNT web-like thin film was produced through CVD, while the solvent-induced condensation process created a void area between the tubes, contributing to the high optical transparency under mechanical strain (Figure 2b) [111]. Furthermore, as the percolation network structures maintain high electrical conductivity even under large mechanical strain, the device shows considerably less impedance change than that of the graphene electrode, even at higher strains (Figure 2c). The cyclic deformation test demonstrates the possibility of long-term electrophysiological recording, because there is no significant change in impedance with up to 6000 cycles of the mechanical fatigue test under 20% tensile or compressive strain. (Figure 2d). With these excellent optical, electrical, and mechanical properties, the in vivo optogenetic stimulation and corresponding neural recordings from Thy1-ChR2-YFP mice were concurrently taken using the transparent electrode arrays. Figure 2e shows the larger amplitude of optical evoked action potentials, with a higher intensity and duration of stimulus by blue laser illumination. The light-induced artifact comparison of the CNT electrode and Au control electrode in saline demonstrates that the potentials induced by the laser stimuli are not light-induced artifacts but evoked potentials by depolarization (Figure 2f).

### 3.2. Graphene-Based Microelectrodes

**Figure 3:**
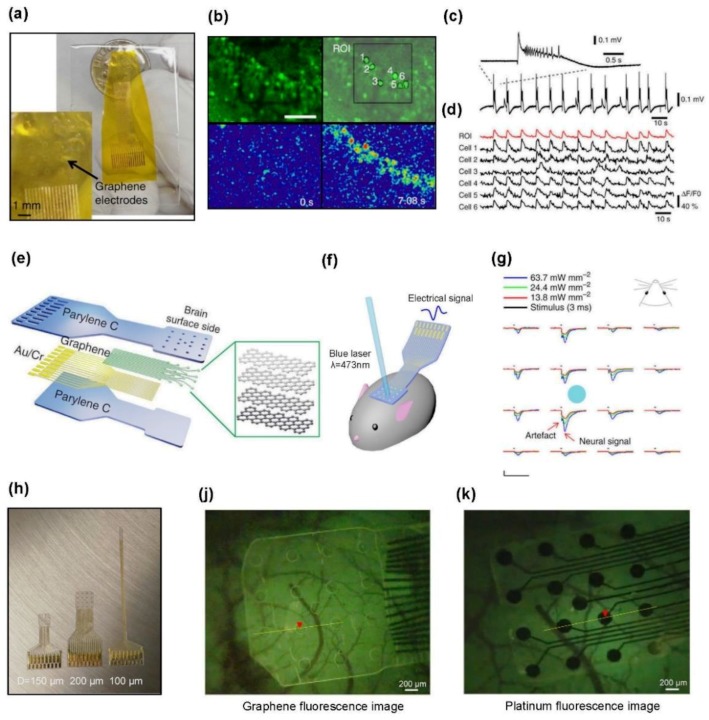

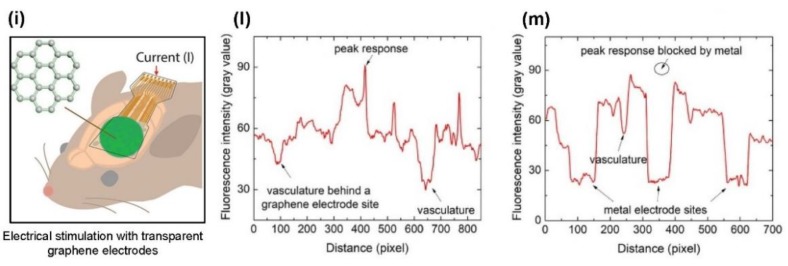
Graphene-based transparent devices for optical imaging and optogenetics: (**a**) photograph of a 16-electrode transparent electrocorticogram (ECoG) array. The electrode size is 300 × 300 μm^2^ each; (**b**) top left: a steady-state fluorescence image of the dentate gyrus in an OBG-1 AM-stained hippocampal slice, where the blue laser and fluorescence emission penetrated through the transparent graphene electrode. Top right: region of interest (ROI) and six random cells within the electrode. Bottom: color-coded images of normalized fluorescence change (ΔF/F0) at the baseline (0 s, left) and at peak response (7.08 s, right); (**c**) time-dependent electrophysiological recording from graphene electrode demonstrates interictal-like activity; (**d**) time-dependent calcium transient (ΔF/F0) for the six cells within the ROI in b. Increases in calcium are generally consistent with interictal-like events in c. Reproduced with permission from reference Kuzum et al. [38], copyright 2014, Macmillan Publishers Limited; (**e**) schematic diagram of carbon-layered electrode array (CLEAR) device consisting of the layered structures; (**f**) schematic illustration of optogenetic testing, where the CLEAR device was implanted on the cortex of a Thyl:ChR2 mouse, with an optical fiber delivering blue laser stimuli to the neural cells; (**g**) average optical evoked responses recorded by the CLEAR device. The x-scale bar, 50 ms; y-scale bar, 100 μV. Reproduced with permission from reference Park et al. [112], copyright 2014, Macmillan Publishers Limited; (**h**) optical camera image of three types of graphene electrode arrays of different diameters (100, 150, and 200 μm); (**i**) schematic drawing of graphene μECoG electrodes implantation and electrical stimulation in GCaMP6f mice. Fluorescence images after the electrical stimulation delivered to the cortex through a single graphene electrode (**j**) and a single platinum electrode (**k**); the stimulation site is marked with a red triangle. Fluorescence intensity over (**l**) graphene electrodes (data from yellow line in j) and (**m**) platinum electrodes (data from the yellow line in k). Reproduced with permission from reference Park et al. [115], copyright 2018, American Chemical Society.

Graphene is another candidate for a transparent flexible electrodes arrays because of its ultrathin geometry with a unique two-dimensional structure and mechanical flexibility. Kuzum et al. presented a transparent, flexible neural interface for simultaneous ECoG and calcium imaging, by transferring CVD-grown graphene electrodes on a polyimide substrate (Figure 3a) [38]. The transparent graphene electrode does not act as an image artifact, blocking the fluorescence of the dentate gyrus in a calcium indicator-stained hippocampal slice when it is excited by a confocal microscope. As a result, the calcium transient mapping for the six randomly selected cells within an electrode site shows a remarkable coincidence with the recorded interictal-like event from the electrode, as shown in Figure 3b–d. The advantage of this single-cell resolution of calcium imaging, combined with the advantage of the temporal resolution of graphene microelectrodes, provides extensive information on neural activities.

The development of transparent graphene electrode arrays enables the optogenetic stimulation of the underlying brain tissue, as in the case of CNTs, as previously described. Park et al. developed a fully transparent and flexible graphene-based carbon-layered electrode array (CLEAR) by coating the graphene electrodes with Parylene C films (Figure 3e) [112]. The fabrication of CLEAR involves the deposition of Au/Cr traces, in addition to the four monolayers graphene. However, the use of these metals is only for the interconnection between the graphene electrodes and the printed circuit board, so it does not affect the transparency of the electrode sites. The transparent CLEAR device can be implanted on the cerebral cortex of a Thy1:ChR2 mouse for optogenetic stimulation (Figure 3f). As shown in Figure 3g, the optically evoked potentials show the increase of amplitudes with an increase in intensity of the laser stimuli, and are divided into an initial peak and a following longer peak. The control experiment, in which mice were killed after the original illumination experiment, revealed that the initial peak was a light-induced artifact and that the second peak was an evoked action potential. 

Microelectrodes are capable of not only recording the neural signals, but also exciting or inhibiting the action potentials by applying an electrical stimulation to neurons [113]. As the electrical current is injected into the neural tissue from the electrodes, the polarity of the neuron membrane changes, which evokes action potentials. There are two mechanisms by which electrical charges are transferred from electrodes to tissue, the capacitive mechanism and the faradaic mechanism [114]. The capacitive mechanism is safer than the faradaic injection is, in that it stimulates by charging and discharging the surface of the electrode without directly injecting charge into the tissue. Park et al. developed another CLEAR transparent electrodes that allow the electrical stimulation current to be delivered to the cortex with a capacitive mechanism, and simultaneously enables optical in vivo monitoring (Figure 3h,i) [115]. The authors compared the capabilities of the CLEAR device and the platinum electrode array during electrical stimulation using transgenic mice with the novel calcium indicator GCaMP6f [89,116]. In the case of CLEAR device, the fluorescence evoked by the electrical stimulation is clearly visible, whereas in the platinum arrays, it is obstructed by the opaque electrodes and interconnects (Figure 3j,k). Figure 3l,m shows the graphical representations of the fluorescence intensity corresponding to the CLEAR device and platinum array, respectively. As the peak response of fluorescence occurs at the electrode site where the electrical pulse is transmitted, no peak response can be observed at the platinum fluorescence intensity.

## 4. CNT/Graphene Based Sensors

### 4.1. Field-Effect Transistors (FET) for Biosensors

#### 4.1.1. CNT Field-Effect Transistors

Since the first demonstration by the Dekker group in 1998 [117], semiconducting single-walled carbon nanotubes (SWCNTs) have been broadly used as channel layers for field effect transistors (FETs). The SWCNT channel has mobility tunable by varying the arrangement of each tube. Kocabas et al. showed that the mobility of a randomly scattered SWCNT network (~10 cm^2^/V·s) is much lower than that of a perfectly aligned SWCNT in one direction (~1000 cm^2^/V·s) [118,119]. Although several postsynthesis strategies aligning SWCNTs in one direction with electric fields [120], magnetic fields [121], or mechanical force [122] have been introduced, the alignment during the growth of the CNTs through the CVD process is the most efficient in terms of fabrication simplicity and high quality of the product [123]. 

The SWCNT FETs have been used extensively for label-free biosensors because the small diameters of SWCNTs match well with the size of biomolecules, such as protein, nucleic acid, antigen, and bacteria [124,125]. A great deal of research has been done on SWCNTs as biosensors. In general, they are proposed to sense biomolecules by two major mechanisms, electrostatic gating and Schottky barrier effects [126,127,128]. However, the fact that these two mechanisms have different gate-voltage dependencies makes it difficult to choose the appropriate gate-voltage. Thus, the Dekker group presented the metal passivation strategy, which inhibits the Schottky barrier effect, making electrostatic gating the dominant mechanism. A more detailed introduction to the mechanisms of biosensing with SWCNT FETs can be found in a report from the Dekker group [129].

Although the hydrophobic nature of the SWCNT surface induces the nonspecific bonding of biomolecules, surface modification through biofunctionalization can lead to selectively immobilizing biomolecules for detection [130]. For example, Kim et al. employed the SWCNT FETs functionalized with various ratios of linkers to spacers for the detection of a prostate cancer marker (PSA-ACT complex) [131]. In this way, they successfully lowered the detection limit to 1.0 ng/mL, without labeling the marker protein. In addition to detecting proteins, Star et al. developed a covalently attached complementary DNA strand (cDNA) on the surface of SWCNT FETs to bind the target single-stranded DNA, resulting in DNA adsorption and hybridization [132]. More information on SWCNT FET biosensors can be found in other reviews [133,134,135].

#### 4.1.2. Graphene Field-Effect Transistors

Among the exceptional properties of graphene, low mechanical stiffness, high optical transparency, and high electrical conductivity are the most noticeable. As the electrical properties of the CNT, in the previous section, rely on the number of layers in the tube, those of graphene also show a similar dependency. The conductivity of graphene decreases gradually with the increasing number of layers by forming graphite. [136]. Bolotin et al. showed that the carrier mobility of a freely suspended monolayer of graphene exceeded 200,000 cm^2^/V·s [137]. In graphene, carriers move as though they are massless fermions. This phenomenon results in a high mobility of graphene that allows significant interest for the applications in next-generation high-speed field-effect transistors [138]. However, although graphene has a high mobility that outperforms other semiconducting materials, the absence of a bandgap in the pristine graphene layer hinders its applications as a semiconductor. In the energy band structure of graphene, there is a region where the valence band and conduction band overlap, but as density of state is zero at that point, the undoped graphene is classified as a semimetal rather than a semiconductor [139]. To this end, various strategies for controlling the bandgap of graphene have been studied and can be found in several review papers [140,141,142].

Like SWCNTs, graphene is very promising material for an active biosensor to detect target biomolecules, because of its combination of properties, including a high conductivity and large surface area. Ohno et al. introduced an FET that uses a nonfunctionalized single layer of graphene as a channel for detecting electrolyte pH and protein concentrations [143]. The conductance of their graphene-based FET (GFET) shows a linear pH dependency and an increasing inclination towards protein adsorption, up to the several hundred picomolar level. Surface functionalization methods involving covalent or noncovalent interactions for SWCNT sensors can also be applied to graphene sensors for the selective adsorption of biomolecules [144]. Mohanty and Berry reported a label-free ssDNA detector using chemically modified graphene with controllable functionality [145]. They suggested that by manipulating the surface functionalization, the sensitivity and polarity of the GFET device can be modified. Another CVD-grown n-doped GFET was fabricated by Dong et al., to detect ssDNA with a high sensitivity of 0.1 nM [146]. Moreover, the outstanding mechanical flexibility of graphene offers potential applications in wearable or implantable electronics. The CVD-grown GFET produced by Kwak et al. was implemented as a flexible glucose sensor by integrating GFET on polyethylene terephthalate (PET) as a substrate [147]. The glucose detection range of the fabricated GFET is 3.3–10.9 mM, which is comparable to that of the conventional screen test used to diagnose diabetes. A perfectly flexible FET sensor based on reduced graphene oxide (rGO) was developed by He et al. to detect fibronectin at a concentration as low as 0.5 mM [148].

### 4.2. Flexible Sensors for Wearable Devices

**Figure 4:**
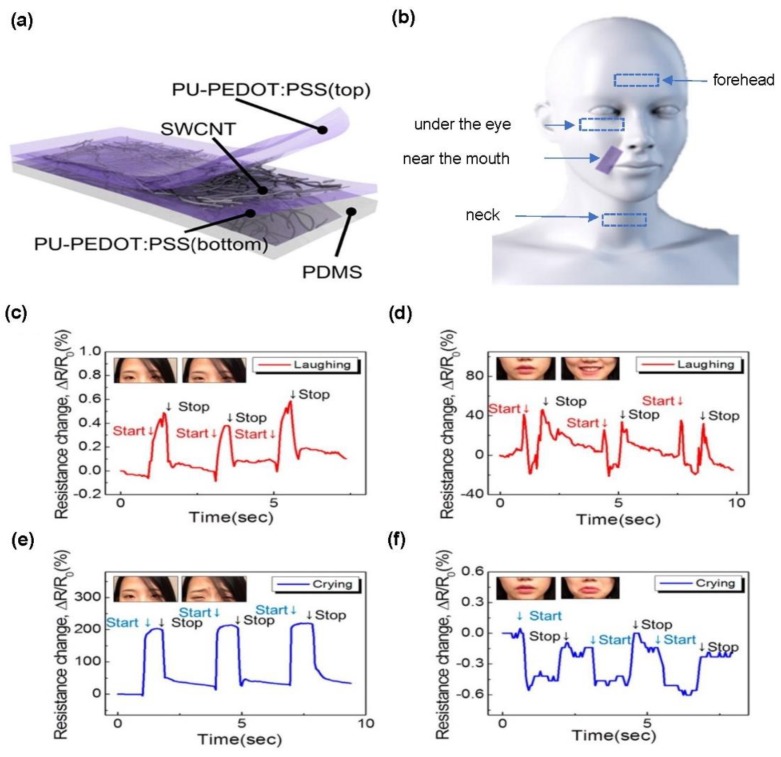
CNT-based strain sensor for facial expression recognition: (**a**) schematic illustration of the cross-section of the strain sensor showing the three-layer stacked structure of the polyurethane poly(3,4-ethylenedioxythiophene) polystyrenesulfonate (PU-PEDOT:PSS)/single-walled carbon nanotubes (SWCNTs)/PU-PEDOT:PSS composite elastomer on a PDMS substrate; (**b**) schematic illustration of stretchable transparent strain sensors attached to four different facial regions to sense skin strains by muscle movements during the expression of emotions; (**c**–**f**) time-dependent ΔR/R0 responses of the sensor attached to (**c**) forehead and (**d**) skin near the mouth with the subject laughing, and of the sensor attached on the (**e**) forehead and (**f**) skin near the mouth with the subject crying. Reproduced with permission from Roh et al. [154], copyright 2015, American Chemical Society.

The CNT or graphene thin films can be assembled with a soft elastomeric substrate, such as PDMS or Ecoflex, to form stretchable and transparent electronics with various functions [149,150]. These carbon-based stretchable devices can be utilized as wearable or implantable bio-integrated sensors, because they can provide conformal contact to the curvilinear shapes of human body. The most important performance factor of a wearable sensor is its sensitivity. Particularly, in the case of a strain sensor, the gauge factor (GF) determines the sensitivity. A variety of elastomeric composites of metals, semiconductors, and carbon nanostructures showed an improved GF value (>15) and stretchability [151,152,153]. However, optical transparency is another important feature of wearable sensors, to enhance the aesthetic effect. For this reason, CNTs and graphene-based wearable sensors with high GF, stretchability, and considerable transparency are attracting attentions. Roh et al. fabricated a transparent and patchable strain sensor by stacking a nanohybrid film of SWCNT and an elastomeric composite of polyurethane (PU)-poly(3,4-ethylenedioxythiophene) polystyrenesulfonate (PEDOT:PSS) on a PDMS substrate (Figure 4a) [154]. Their sandwich-like piezoresistive strain sensor, in which SWCNT nanofillers are confined in a conductive elastomeric matrix, have a gauge factor of 62, which outperforms conventional metallic or carbon-based strain sensors [155,156,157]. These highly sensitive strain sensors can be conformably attached to several parts of the face, sensing the change of facial expression due to muscle movement or wrinkling of the skin (Figure 4b). The strain sensors onto the forehead and skin near the mouth clearly distinguish between the subject’s laughing and crying. Normally, when a person laughs, the movement of the mouth is larger than that of the other facial regions, while the movement of the forehead becomes remarkable when crying. Figure 4c–f demonstrates the quantified results from the resistance change of the strain sensors attached on forehead and near the mouth.

**Figure 5:**
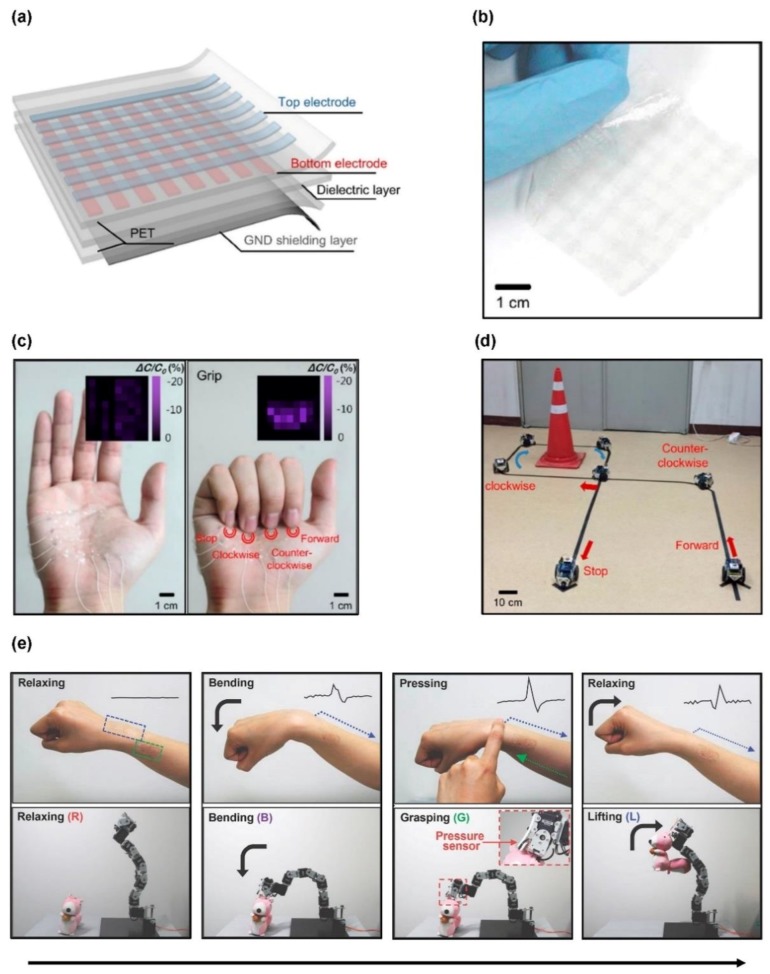
Graphene-based wearable sensors for a human-machine interface: (**a**) schematic illustration of a graphene-based capacitive sensor consisting of two graphene electrodes layers and an intermediate dielectric layer; (**b**) optical camera image of 64-channel touch sensor array; (**c**) images of stretchable touch sensor mounted on a palm for remote control application. Inset: capacitance changes for spread (left) and grip (right) status of the palm; and (**d**) images of a toy car operated by the stretchable remote control in c. Reproduced with permission from Kang et al. [158], copyright 2017, American Chemical Society. (**e**) The transparent motion sensor and the electrotactile stimulator attached on the wrist and forearm, respectively. The bending, pressing, and relaxing of the wrist lead to the bending, grasping, and lifting of the robot arm, respectively. Reproduced with permission from Lim et al. [160], copyright 2014, WILEY-VCH.

Capacitive touch sensors are sometimes preferred over resistive sensors, because of their capabilities of multitouch sensing. Kang et al. presented a graphene-based stretchable touch sensor capable of multitouch sensing and three-dimensional sensing for the recognition of 3D shapes [158]. These touch sensors consist of graphene top and bottom electrodes transferred onto an ultrathin polyethylene terephthalate (PET) substrate, an intermediate dielectric layer, and a monolayer graphene for bottom grounding (Figure 5a). The completed sensor array has a transmittance of more than 80% in the visible range, and its thin structure and stretchability allow conformal contact to the curved surface (Figure 5b). The stretchability is further improved when the auxetic mesh-like structure is applied to the device [159]. The touch sensor array of the auxetic structure was attached to the palm with conformal contacts and used to remote control the movement of a toy car through simple finger motions (Figure 5c,d).

Another example of a carbon-based transparent and stretchable motion sensor was introduced by Lim et al. [160]. Their stretchable and wearable piezoelectric motion sensors and electrotactile stimulators based on a graphene heterostructure are utilized as an interactive human­–machine interface (iHMI) that bidirectionally connects human and machines. In the iHMI system, motion sensors convert human motion into electrical signals that control the machine, and the electrotactile stimulators deliver feedback signals from the machine back to the human body. The graphene heterostructure can be used in both sensors and stimulators of the iHMI system. The motion sensor consists of a heterostructure with a piezoelectric polymer/SWCNT composite film sandwiched between two layers of CVD-grown graphene and polymethylmethacrylate (PMMA) insulating layers. The SWCNTs are embedded in polylactic acid (PLA), which is a piezoelectric polymer [162], to enhance SNR. The electrotactile stimulator consists of a heterostructure containing silver nanowire (AgNW) networks embedded in graphene layers, epoxy encapsulation layers, and a PDMS substrate. The stretchable iHMI system attached to the human arm can interactively control the robot arm. Through a data acquisition board and a special software program, the robot arm identified several movements of the human arm and performed the corresponding actions (Figure 5e). Furthermore, electrical stimulation can provide feedback to the user to prevent excessive operation of the robot arm. 

**Figure 6:**
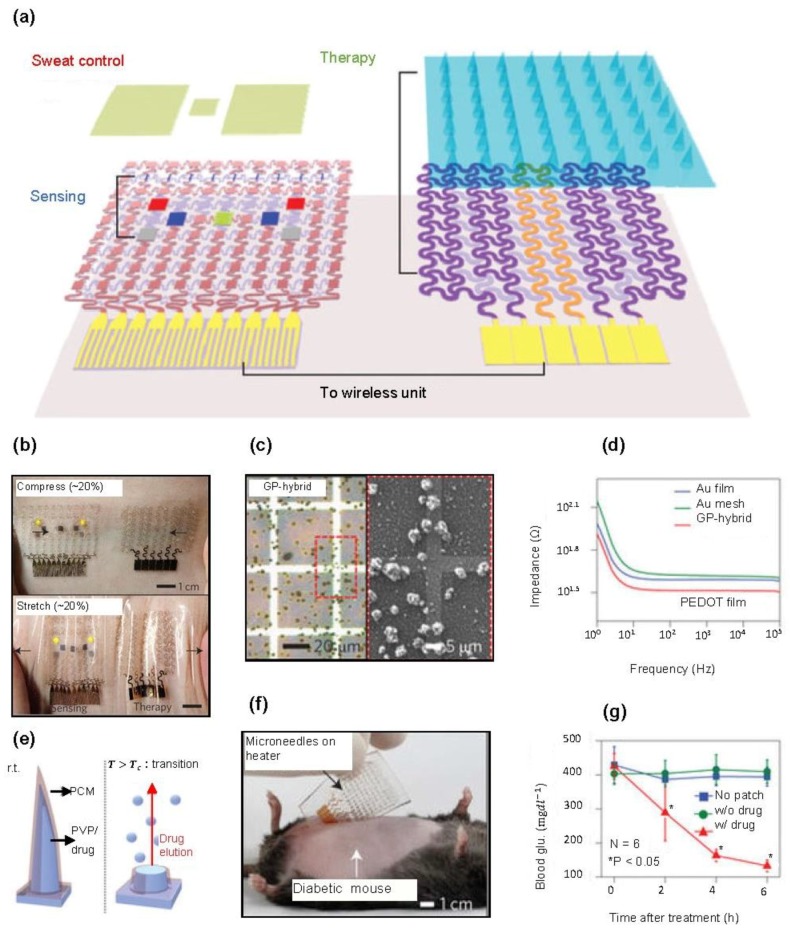
Graphene-based electrochemical sensors and microneedles for diabetes monitoring and therapy: (**a**) schematic illustration of the diabetes patch system consisting of sweat control, sensing (humidity, glucose, pH, and tremor sensor) and therapy components (heater, temperature sensor, and microneedles with drugs) on a silicone substrate; (**b**) photographs of the diabetes patch attached on the human skin under 20% applied compressive (top) and tensile (bottom) strain; (**c**) optical microscopy (left) and SEM (right) images of the graphene-hybrid with the PEDOT electrodeposition; (**d**) bode plots of the three electrodes in PBS after PEDOT electrodeposition; (**e**) schematic illustrations of the thermally active bioresorbable microneedles; (**f**) optical camera image of the therapeutic patch with microneedles on heater laminated on the skin near the abdomen of the diabetic (db/db) mouse; and (**g**) blood glucose concentrations of the db/db mice for the experimental group and control groups. The error bars and asterisks indicate the standard deviation in each group and significant difference (*p* < 0.05) among the groups at each time point relatively. Reproduced with permission from Lee et al. [161], copyright 2016, Macmillan Publishers Limited.

Lee et al. have developed a patchable graphene-based device that can simultaneously diagnose and treat diabetes by combining various electrochemical sensors, including humidity, glucose, pH, tremor, and temperature, with thermoresponsive microneedles for drug delivery on stretchable silicone substrate (Figure 6a) [161]. Their multifunctional electrochemical device integrated on soft materials and serpentine design provides extremely conformal contact to the skin even under 20% tensile or compressive strains (Figure 6b). In addition, the GP-hybrids, consisting of a bilayer of Au mesh and gold-doped graphene, facilitate surface functionalization via electrodeposition, providing better electrochemical properties (Figure 6c,d). The high glucose concentration detected by the sensing part activates the heater in the therapy part, and the heat generated thereby dissolves the microneedles containing the drug to perform drug delivery into the blood (Figure 6e). As shown in Figure 6f,g, lamination of the therapeutic patch onto the abdomen of diabetic rats resulted in significantly reduced blood glucose levels, compared to those of the control groups.

## 5. Conclusions

This review covers some of the latest bioelectronics based on carbon nanotubes and graphene, the most representative carbon nanomaterials. In addition to their high electrical conductivity and optical transparency, excellent mechanical properties that are not found in conventional metal or semiconducting materials have allowed them to be extensively studied as wearable or implantable electronics. However, even though those physical properties of CNTs and graphene are outstanding, they cannot be integrated into the human body if their definite biocompatibility is not established. CNTs and graphene show varying biocompatibility depending on their concentration, method of synthesis, functionalization, and the type of cells to which they are applied. Although most studies using carbon nanomaterials in mammalian cells have shown biocompatibility, careful consideration of the application types is needed, as there are some studies that show toxicity to certain tissues, such as lung and skin. We have introduced biocompatible and flexible CNT/graphene-based bioelectronics that can be applied in epidermal or implantable electronics in two categories, according to their application, namely, microelectrodes and sensors.

The surface coating of traditional microelectrodes with CNTs or composites of CNTs and other conductive materials, including PEDOT and gold, remarkably increases the surface roughness, thereby resulting in a high SNR. Furthermore, the high optical transmittance of graphene and CNT thin films enables optical imaging and optogenetic stimulation, at the same time as electrical recording, offering high temporal and spatial resolution. CNTs and graphene with tunable bandgaps are suitable for sensors that detect biomolecules, such as proteins and nucleic acids, with a high sensitivity due to their high mobility and surface area. Recent studies have developed a novel human–machine interface that converts human facial expressions and movements into electrical signals using carbon-based piezoresistive, capacitive, and piezoelectric strain sensors. The integration of various electrochemical sensors and microneedles for drug delivery presents a new scheme that enables the diagnosis and treatment of hyperglycemia with a thin and transparent graphene-based patch.

These technological advances in carbon-based materials have contributed greatly to the realization of neural mapping, diagnosis and treatment of various diseases, and to the human–machine interface. Nevertheless, there are still unsolved issues that must be addressed to make these applications even better. Although CNTs and graphene have electrochemical properties that surpass other conventional electrodes for neural interfaces, there are still size-related problems in measuring the activity of all single neurons. To be integrated with a highly stretchable substrate for a more conformal interface with the human body, improved manufacturing technologies, such as low-temperature processes or transfer processes, are required. Furthermore, although many examples of implantable devices have been introduced, there is still a lack of research on devices for long-term usage in the biological environments. For example, CNTs and graphene can be oxidized and peeled off from the electrode surfaces under the electrical stimulation [163]. Establishing complete biocompatibility and creating fully biodegradable electronics, combined with the proper encapsulation layers is another challenge requiring solutions in the future [164]. Continued research on carbon-based materials is expected to overcome these challenges and make an important impact on human healthcare.
